# Assessment of Retrieved N_2_O, NO_2_, and HF Profiles from the Atmospheric Infrared Ultraspectral Sounder Based on Simulated Spectra

**DOI:** 10.3390/s18072209

**Published:** 2018-07-09

**Authors:** Hongmei Wang, Xiaoying Li, Jian Xu, Xingying Zhang, Shule Ge, Liangfu Chen, Yapeng Wang, Songyan Zhu, Jing Miao, Yidan Si

**Affiliations:** 1 State Key Laboratory of Remote Sensing Science, Institute of Remote Sensing and Digital Earth, Chinese Academy of Sciences, Beijing 100101, China; wanghm@radi.ac.cn (H.W.); chenlf@radi.ac.cn (L.C.); wangyp@radi.ac.cn (Y.W.); soonyenju@outlook.com (S.Z.); miaojing16@mails.ucas.ac.cn (J.M.); siyd@radi.ac.cn (Y.S.); 2 University of Chinese Academy of Science, Beijing 100049, China; 3 Remote Sensing Technology Institute, German Aerospace Center (DLR), Oberpfaffenhofen, 82234 Weßling, Germany; jian.xu@dlr.de; 4 National Satellite Meteorological Center, China Meteorological Administration, Beijing 100081, China; zxy@cma.gov.cn; 5 China Center for Resources Satellite Data and Application, Beijing 100094, China; gslcresda@126.com

**Keywords:** GF5 AIUS, occultation, retrieval algorithm, microwindow selection, N_2_O, NO_2_, HF

## Abstract

The Atmospheric Infrared Ultraspectral Sounder (AIUS), the first high-resolution (0.02 cm^−1^) solar occultation sounder, aboard GF5, was launched in May 2018 from China. However, relevant studies about vertical profiles of atmospheric constituents based on its operational data were not conducted until half a year later. Due to an urgent need for Hin-orbit tests, the real spectra (called reference spectra hereafter) were substituted with simulated spectra calculated from the reference forward model (RFM) plus different random noises at different altitudes. In the generation process of the reference spectra for N_2_O, NO_2_, and HF species, ACE-FTS (Atmospheric Chemistry Experiment–Fourier Transform Spectrometer instrument on the SCISAT satellite) level 2 products replace corresponding profiles included in the atmospheric background profiles. The optimal estimation method is employed to extract N_2_O, NO_2_, and HF profiles in this study. Comparing the retrieved results with ACE-FTS level 2 products, the relative deviations for these three species are calculated. For N_2_O, the average relative deviation is less than 6% at altitudes below 25 km, while larger deviations are observed in the range of 25–45 km, with the maximum being at ~25%. Additionally, the difference for NO_2_ is less than 5% in the 20–45 km range, with a larger discrepancy found below 20 km and above 45 km; the maximum deviation reaches ±40%. For HF, the relative deviation is less than 6% for all tangent heights, implying satisfactory retrieval. The vertical resolution, averaging kernel, and number of degrees of freedom are used to assess the retrieval algorithm, which indicate that the retrieved information content is much more attributable to the reference spectra contribution than to the a priori profile. Finally, a large number of retrieval tests are performed for N_2_O, NO_2_, and HF in selected areas covering the Arctic region, northern middle latitude, tropics, southern middle latitude, and Antarctic region, and reliable results are obtained. Thus, to a great extent, the algorithm adopted in the AIUS system can process retrievals reliably and precisely.

## 1. Introduction

The Atmospheric Infrared Ultraspectral Sounder (AIUS) has a high spectral resolution of 0.02 cm^−1^ while operating over a broad wavenumber range of 2.4–13.3 µm (750–4100 cm^−1^). AIUS was designed to measure atmospheric absorption spectral sequences. These spectra, detected in the limb viewing geometry tangent heights, are inverted to obtain the vertical profiles of atmospheric constituents [1]. The goal of AIUS is planned to measure the profiles of gases, such as O_3_, H_2_O, CO, HNO_3_, NO, NO_2_, N_2_O, HCl, and HF, in the troposphere, stratosphere, mesosphere, and partial thermosphere above and surrounding Antarctica (50° S–90° S). This paper aims to is to test the retrieval performance by using synthetic measurements based on AIUS instrument characteristics for HF, NO_2_, and N_2_O. AIUS, which was launched on 9 May 2018, features instrument parameters that are similar to those of ACE-FTS. Therefore, three ACE-FTS level 2 profile products were assumed as the true profiles to simulate the spectra of AIUS for HF, NO_2_, and N_2_O. The Atmospheric Chemistry Experiment (ACE) was launched successfully into orbit on 12 August 2003, with a high inclination (74°) at 650 km [2]. Its primary instrument is a high-spectral-resolution (0.02 cm^−1^) infrared Fourier transform spectrometer (ACE-FTS), with a wavenumber range of 750–4400 cm^−1^.

NO_2_ and other nitrogen oxide constituents, commonly referred to as NO_x_, are highly influential in atmospheric chemistry. As a precursor of photochemical smog, the chemical reaction of NO_2_ with hydrocarbons is the main factor accounting for surface ozone pollution, which cannot be ignored in air pollution. The NO_x_ gas phase catalytic cycle destroys odd oxygen in the stratosphere, while NO_2_ also plays a prominent role in determining the polar ozone budget [3]. The primary sources of NO_x_ are fossil fuel combustion, vehicle emissions, biomass burning, lightning, and soil emissions, with anthropogenic emissions accounting for a substantial proportion of the total emissions. Nitrous oxide (N_2_O) is the major source gas for nitrogen oxides in the stratosphere, a useful dynamical tracer, and one of the three main greenhouse gases (CO_2_, CH_4_, and N_2_O). N_2_O is of surface and near-surface origin, with approximately equal contributions from natural and anthropogenic emissions. As the only long-lived atmospheric tracer of human perturbations of the global nitrogen cycle [4], tropospheric N_2_O is transported through the tropical tropopause into the stratosphere, where approximately 90% is photolyzed in the wavelength range of 185–220 nm, generating N_2_ and O. The remaining 10% is decomposed by the reaction with O(^1^D) [5]. HF, which is long lived in the stratosphere, is often used as a simple reference to other column observations of chemically active stratospheric components, such as HCl. The unique known sink of HF is transported to the troposphere, after which it is cleaned up by rainfall. The long lifetime of HF keeps fluorine chemistry from being a significant sink for stratospheric ozone. Therefore, this gas is a useful tracer of stratospheric motion and is often used as a reference for other chemically active tracers [6].

Remote sounding of the Earth’s limb is currently the only method of observing the profiles of atmospheric constituents from the upper troposphere to the lower thermosphere. Many trace gases have their own signature in infrared bands, which are used to retrieve profiles by infrared occultation and limb sounders. Much of the research on N_2_O, NO_2_, and HF has been conducted using other occultation and limb instruments. ATMOS (Atmospheric Trace Molecule Spectroscopy), on board the Space Shuttle, was a Fourier transform infrared spectrometer. ATMOS operated on a broad band range of 600–4800 cm^−1^ and could provide information about the chemical composition of the atmosphere, such as CO_2_, O_3_, CH_4_, N_2_O, CO, and H_2_O in the troposphere, stratosphere, and mesosphere [7]. Analyses of the retrieval volume mixing ratios (VMRs) of N_2_O have been carried out through the middle atmosphere using 0.01-cm^−1^ resolution infrared solar occultation spectra, which were recorded near 28° N and 48° S latitudes with ATMOS and were compared with results from other ground-, balloon-, and satellite-based instruments [8]. HALOE (halogen occultation experiment) is carried on the high-atmospheric research satellite UARS (Upper Atmosphere Research Satellite), launched by NASA on 12 September 1991. The scientific goal of HALOE (2.45–10.04 µm) is to improve the understanding of the stratospheric ozone depletion related to ClO_y_, NO_y_, and HO_y_ by collecting and analyzing global data of key chemical components (O_3_, HCl, NO, NO_2_, HF, CO_2_, etc.) while simultaneously observing the effects of fluorine on ozone [9]. Measurements of NO_2_ and HF are detailed and validated from HALOE in published papers [10,11]. ACE-FTS can be used to retrieve the vertical distribution of more than 30 kinds of atmospheric components, including O_3_, H_2_O, HCl, N_2_O, CO, CH_4_, NO, NO_2_, and HF, from the surface to 120 km, and to study the atmospheric chemical and dynamic processes of the ozone distribution in the upper troposphere. VMR profiles of HF, N_2_O, and NO_2_ were retrieved from ACE-FTS, and the quality of ACE-FTS version 2.2 data was evaluated using other solar occultation measurements, including HALOE, SAGE II, SAGEIII, POAMIII, SCIAMACHY, stellar occultation measurements (GOMOS), and limb measurements (MLS, MIPAS, and OSIRIS) [2,3,5,12,13]. MIPAS (Michelson Interferometer for Passive Atmospheric Sounding) is a limb-viewing Fourier transform spectrometer that sounded the emission of Earth’s atmosphere covering the spectral range from 685 to 2410 cm^−1^ aboard the ESA (European Space Agency) Envisat satellite [14]. The ESA operational almost real-time analysis provided vertical profiles of temperature and of VMR of H_2_O, O_3_, HNO_3_, CH_4_, N_2_O, and NO_2_ from pole to pole, approximately following the orbital track [14]. The TES (Tropospheric Emission Spectrometer) is an instrument on the EOS Aura platform designed to study the Earth’s ozone, air quality, and climate. TES measured the spectral infrared (IR) radiances (650–3050 cm^−1^) in a limb-viewing and a nadir (downward looking) mode. The scientific goal of TES is to detect the tropospheric O_3_, CH_4_, NO_2_, NO, HNO_3_, CO, and H_2_O related to tropospheric O_3_ chemical reactions [15,16]. Due to the high resolution and broad band range of AIUS, N_2_O, NO_2_, and HF profiles are retrieved and provide information for subsequent algorithms.

This manuscript is organized as follows. First, an overview of the optimal estimation method (OEM) and diagnostic tools, microwindow selection, atmospheric profiles and a priori profile, and forward model are introduced in Section 2. Comparisons between the observed spectra and the simulated spectra and the retrieval process, in addition to the retrieval results and their assessment, are presented in Section 3. A large number of simulation retrieval tests were conducted, and the results are analyzed in Section 4. Finally, a summary and conclusions are offered in Section 5.

## 2. Retrieval Scheme of N_2_O, NO_2_, and HF

Two crucial factors should be considered before retrieval: the pressure/temperature (P/T) and the tangent point. Reliable knowledge about atmospheric pressure and temperature is essential for the retrieval of VMR profiles [17]. Consideration of the horizontal temperature gradients improves the retrieval accuracy. In addition, in many cases, such consideration reduces the number of convergence failures. In particular, near the polar vortex boundary, many retrievals have failed to converge when horizontal temperature gradients were neglected [18]. Another crucial aspect of the P/T and trace gas retrieval process is pointing knowledge. The tangent height correction is determined before the retrieval of temperature and other trace gas profiles. The other two relevant issues for temperature retrieval and tangent height correction are prepared in advance. In this paper, the impacts of errors in temperature and tangent height on the inversion results are preliminarily evaluated.

### 2.1. Retrieval Algorithm

The reference forward model (RFM) is the forward model employed in the AIUS retrieval algorithm. The latest release version is v 4.36 (http://eodg.atm.ox.ac.uk/RFM/index.html). The RFM can be applied for various measurement conditions, including nadir viewing, limb sounding, occultation observation, and balloon/aircraft measurements [1]. The retrieval problem in atmospheric occultation sounding is how to extract the vertical profiles of the atmospheric state parameters from a sequence of the spectra of different tangent heights. The inversion algorithm adopted in this study is based on the OEM proposed by Rodgers [18,19]. The inversion method used in this study is adapted and modified according to the Qpack 2.0 retrieval software [20]. Details regarding the retrieval algorithm are reported elsewhere [1,20,21]. The retrieval algorithm is expressed as follows:

(1) $$X_{i+1}=X_{i}+{\left[\left(1+\gamma \right)S_{a}^{-1}+K_{i}^{T}\cdot S_{e}^{-1}\cdot K_{i}\right]}^{-1}*\left\{K_{i}^{T}S_{e}^{-1}\left[Y-F\left(X\right)\right]-S_{a}^{-1}\left[X_{i}-X_{a}\right]\right\},$$

where Y is a sequence of observations; *F* denotes the forward model; X is the true state of the atmosphere and $X_{a}$ represents a priori knowledge with an associated covariance matrix $S_{a}$; *S_e_* is the covariance matrix of the observation error; and *K* is the weighting function matrix. Here, γ is not a fixed value related to atmospheric species, and the initial value is taken from an experimental result. In the iterative process, the constraint factor γ must be adjusted and updated.

Considering the correlation between the different components of the state vector and the forward model vector, the definition of the a priori covariance matrix $S_{a}$ can follow three functional types: Gaussian statistics, exponential, and linear form [21]. Based on the retrieval test from the simulated data, the correction function employed in this study is the linear case, which can be expressed as follows:

(2) $$S_{a}\left(i,j\right)\;=\;\max\{0,\;\sigma \left(i\right)\delta \left(j\right)\left[1-\left(1-e^{-1}\right)\frac{2\left|z\left(i\right)-z\left(j\right)\right|}{l_{c}\left(i\right)\;+\;l_{c}\left(j\right)}\right]\}$$

where *i* and *j* denote the position of covariance matrix $S_{a}$; δ is the standard deviation, calculated from a priori knowledge; *z* is the position; $l_{c}$ is the correction length; and |.| implies the absolute value.

Following Rodgers’ method, several powerful diagnostic tools were considered and were used as described below to assess the retrieval approach: the covariance matrix of the retrieved solution, averaging kernel matrix, vertical resolution, and degrees of freedom (DOFs). The covariance matrix of the solution includes the estimated error of the state parameters and the interlevel correlations and is given by

(3) $$\;S_{x}={\left(S_{a}^{-1}+K^{T}S_{e}^{-1}K\right)}^{-1}$$

The vertical averaging kernel is the derivative of the retrieved profile with respect to the true profile [12], which implies the weight of the true atmospheric state. For the optimal estimation formula, the averaging kernel matrix A is calculated as

(4) $$A=\;\frac{\partial \hat{x}}{\partial x}\;=\;\frac{\partial \hat{x}}{\partial y}\frac{\partial y}{\partial x}\;=\;G_{y}K=(K^{T}S_{e}^{-1}{K\;+\;S}_{a}^{-1}{)}^{-1}K^{T}S_{e}^{-1}K$$

where $\hat{x}$ is the retrieved profile and $G_{y}$ is the gain matrix, which describes the sensitivity of the retrieval to the changes in the measurement. In the absence of a constraint in the least-squares problem (2), the averaging kernel matrix A is the identity matrix I (A=I). The vertical resolution of the retrieval as a function of A can be defined in several ways. Here, the vertical resolution is defined as the width at the half-maximum of the column of the averaging kernel matrix A. Due to regularization, the vertical resolution is typically wider than the tangent altitude spacing and is invariably wider than the grid on which the retrieval is performed [18].

According to the trace of the averaging kernel, the number of DOFs is defined as

DOFs = tr(A)(5)

where the DOFs can be represented as the trace of the averaging kernel. If the DOFs is close to the dimension of the retrieving state vector, the retrieval result is determined by measurement information rather than by a priori knowledge.

### 2.2. Microwindow Selection

**Figure 1:**
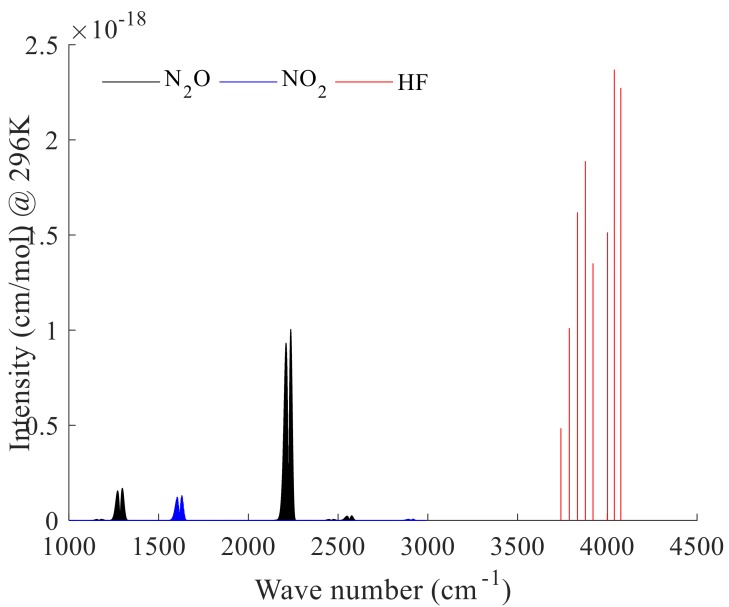
N_2_O, NO_2_, and HF line intensities and distributions.

**Figure 2:**
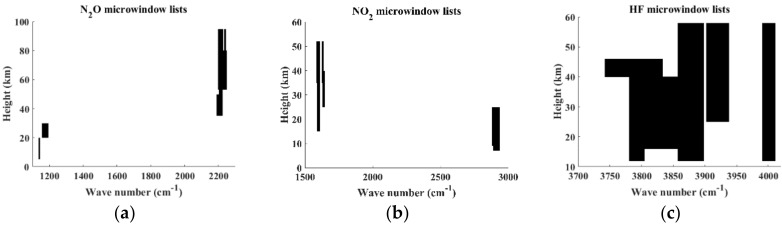
Microwindow lists. (**a**) For N_2_O; (**b**) for NO_2_; (**c**) for HF.

AIUS, a high-spectral-resolution spectrometer, has a strong correlation within channels and a similar spectral resolution and wavenumber range to ACE-FTS. Up to 69 microwindows are used in version 2.2 ACE-FTS for N_2_O retrievals [5], 11 microwindows are selected in version 3.5 ACE-FTS retrievals for HF, and 21 microwindows are employed for NO_2_ retrievals [3]. Fewer and narrower channels and microwindows, respectively, were adopted in this work relative to ACE-FTS for the retrieval of the N_2_O, NO_2_, and HF profiles. This simplification was implemented to improve computational efficiency while maintaining acceptable accuracy. Wavenumber ranges of 1120–1300 and 2200–2250 cm^−1^ for N_2_O, 1560–1642 and 2890–2940 cm^−1^ for NO_2_, and 3700–4110 cm^−1^ for HF were selected based on these considerations. Figure 1 presents the intensity and distribution of the N_2_O, NO_2_, and HF absorption lines at a temperature of 296 K. Microwindow selection was performed based on the sensitivity analysis for interference molecules and target composition and the entropy of information principles according to the set weight function threshold. The Shannon information content is a scalar quantity which is defined qualitatively as the factor (in bits). The microwindows selected for N_2_O, NO_2_, and HF are shown in Figure 2. The *x*-axis indicates the position of the microwindows in the spectral band, and the *y*-axis represents the retrieval information at different tangent heights obtained from different microwindows. Finally, 126, 184, and 283 channels were selected for NO_2_, N_2_O, and HF, respectively. Number of channels (NC) and information content (IC) between ACE-FTS and simulating AIUS were computed and compared following equations of information content [19], which are shown in Table 1. ACE FTS used 2040 channels with 6.92 bits of IC and simulating AIUS used 184 channels with 6.3 bits of IC for N_2_O retrieval. That is to say, simulating AIUS used 9% channels and retained 88% of the IC compared with ACE-FTS. Similarly, simulating AIUS has 6.69 bits of IC for NO_2_ and 6.43 bits of IC for HF, respectively, corresponding to 7.38 bits of IC for NO_2_ and 6.84 bits of IC for HF of AIUS. The probations of AIUS/ACE-FTS were 90% and 94% accordingly.

Small regions of the spectrum (generally 0.02 cm^−1^ for AIUS) contain the spectral features from a target molecule with minimal spectral interference from other molecules. However, for some molecules, it is impossible to find a comprehensive set of microwindows unaffected by significant interference. The following interference molecules were considered: H_2_O, O_3_, CH_4_, CH_3_Cl, CH_3_OH, H_2_CO, CO_2_, ClO, HCl, HNO_3_, NH_3_, N_2_O, NO, and OCS for NO_2_; CO_2_, CH_4_, H_2_O, H_2_O_2_, O_3_, CO, H_2_CO, HNO_3_, NH_3_, and SO_2_ for N_2_O; and O_3_, H_2_O, CO_2_, OCS, CH_4_, and N_2_O for HF.

### 2.3. Atmospheric Profiles

Profiles of the atmospheric species demonstrate a discrete atmospheric state at a specific time in a unique location. An integrated atmospheric profiles algorithm was proposed for the AIUS retrieving system that consists of ACE-FTS level 2 products, MLS global atmospheric products for the last 5 years, and profiles from the AFGL (Air Force Geophysics Laboratory) atmospheric models. The integrated profiles of AIUS start from the surface with a grid width of 1 km and reach upward to as high as 120 km [1].

Rodgers [18,19] proposed the OEM based on the Bayesian theory, introducing an a priori profile to define the range of solutions. The reasonable selection of the a priori profiles and the correct estimation of the a priori error strongly influence the stability, speed, and accuracy of calculations, especially for trace species such as N_2_O, NO_2_, and HF. Therefore, a series of a priori profiles were constructed based on the level 2 products of ACE-FTS (https://databace.scisat.ca/level2/ace_v3.5_v3.6/FLAG/), named ACEFTS_L2_v3p6_*.nc, where * is the name of the target species, for example, O_3_. The a priori profiles for N_2_O, NO_2_, and HF were used to regularize the retrieval and to contribute structure to the retrieval that was not actually measured. The following steps were carried out to build the database of the a priori profiles.

1. Profiles from file ACEFTS_L2_v3p6_*.nc are retrieved and classified by month and further divided by the latitude and longitude spacing of 5° and 30°, respectively.
2. By eliminating the invalid values of the profiles that fall in the same grid and calculating mean values, files for the a priori profiles are generated. We call these the database of the fine grid profiles.
3. Due to the incomplete coverage area of the ACE-FTS products, not all grids have an a priori profile. In view of this situation, another approach is adopted. Due to the coverage of AIUS around the Antarctic (50–90° S), profiles in this region are collected, processed by season, and averaged. We call this set of the obtained a priori profiles a database of the coarse grid profiles that are used when the fine grid profiles are lacking.

Following the date and location of inversion, three a priori profiles of the a priori data from the database of fine grid profiles were selected for HF, N_2_O, and NO_2_ inversion in this study and were then interpolated onto the retrieval grid.

## 3. Results

### 3.1. Simulated Spectra and Reference Spectra

The retrieval scheme of AIUS is based on the RFM to simulate infrared atmosphere radiation transmission. There are two sounding channels: MCT and InSb. MCT covers the band range of 750–1850 cm^−1^ and InSb covers the range of 1850–4160 cm^−1^. The simulated spectra and the reference spectra have locations and dates identical to those of ACE-FTS level 2 products, that is, the orbits are sunrise 40993, 43544, and 48359.

**Figure 3:**
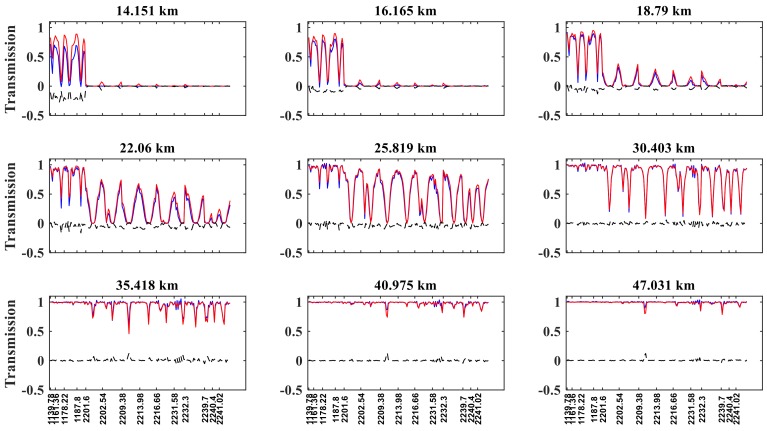
Reference transmission (blue curve), simulated transmission (red curve), and the difference between the real and simulated transmissions (black curve) for different tangent heights in N_2_O retrieving channels, by reference to sunrise orbit 40993 of ACE-FTS. The *x*-axis represents the wavenumber; the *y*-axis represents transmission.

Figure 3 shows that the reference transmission and simulated transmission match well, especially at tangent heights above 16 km. However, for altitudes at 14.151 km, a larger transmission difference occurs at selected channels ranging from 1135 to 1180 cm^−1^, where the N_2_O absorption is weak, with a maximum difference of 0.25. For tangent heights above 40 km, the differences between the real and simulated transmission values are approximately 0 because the N_2_O content decreases with increasing tangent height.

**Figure 4:**
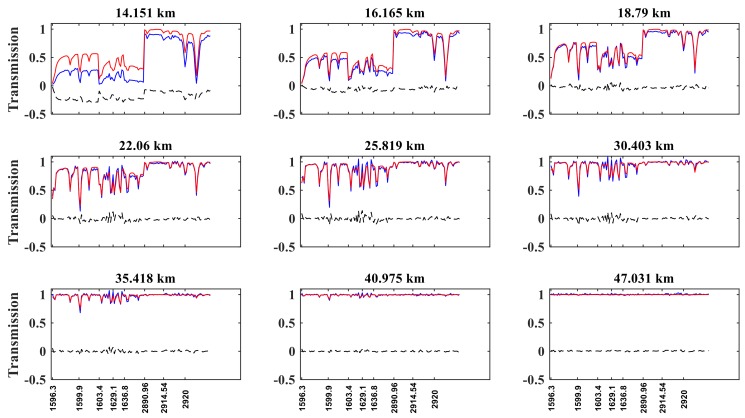
The same as Figure 3 but for NO_2_.

Figure 4 shows the reference transmission, the simulated transmission, and their difference for NO_2_. For tangent heights below 50 km, the two real and simulated transmission values show excellent agreement, whereas for the tangent height of 14.151 km, the difference can reach 0.25.

**Figure 5:**
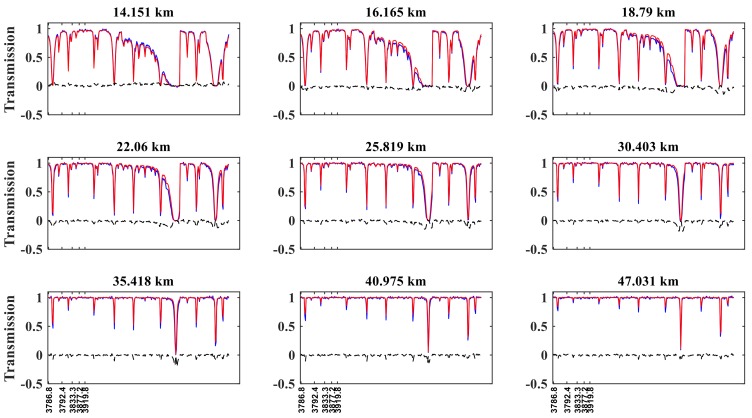
The same as Figure 3 but for HF.

The differences between the reference and simulated transmission values for HF are quite small as shown in Figure 5. The maximum difference does not exceed 0.1, with a relatively large difference at the strong absorption channels. Therefore, the retrieval method described in the next section obtains better results at all tangent heights.

### 3.2. Assessments of Effects of Temperature and Tangent Height on Retrieval Results

On the basis of the NO_2_ microwindows, retrieval algorithm and sunrise orbit 43544 profiles of the ACE-FTS level 2 NO_2_ product, spectra were simulated by temperature plus 1 K, 2 K, and 3 K and tangent height plus 500 m and minus 500 m. The variation in spectra caused by the changes in temperature and tangent height are first discussed, and then the effects on retrieval results of NO_2_ are evaluated.

**Figure 6:**
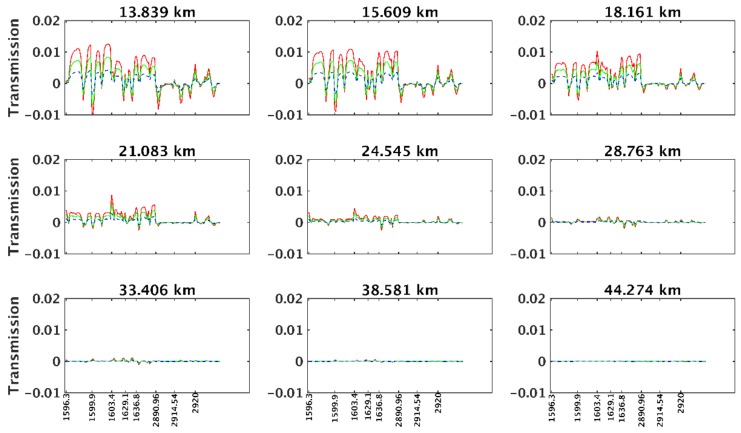
Curves of transmission variation caused by temperature plus 1 K (blue curve), 2 K (green curve), and 3 K (red curve) at different tangent heights.

Figure 6 shows the variations in transmission caused by temperature plus 1 K, 2 K, and 3 K. It can be seen that a change in temperature can lead to an obvious change in transmission. With the increase in temperature, the change in transmission becomes more significant. The major influence of temperature variation on spectra occurs below 20 km, with a maximum transmission variation of 0.01 caused by increasing the temperature by 3 K. Above 20 km, the transmission variation caused by temperature change becomes less obvious. Thus, the temperature mainly affects the absorption ratio of atmospheric species below the lower stratosphere.

**Figure 7:**
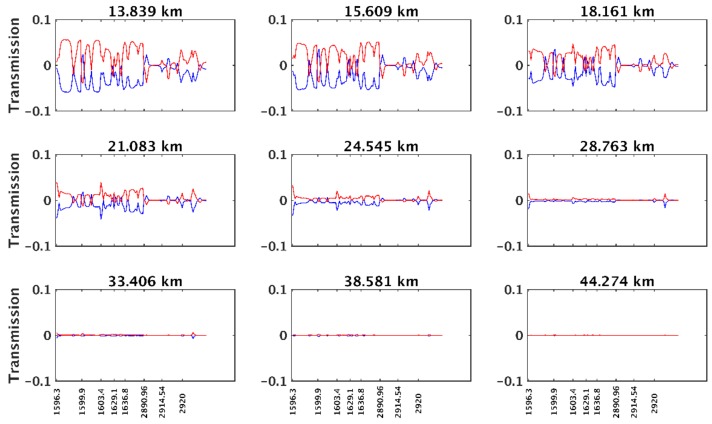
Curves of transmission variation caused by tangent height plus 500 m (red curve) and minus 500 m (blue curve) at different tangent heights.

Figure 7 shows the variations in transmission caused by tangent heights plus 500 m and minus 500 m. The red curve is the transmission difference between the testing tangent height plus 500 m and the testing tangent height (as shown in the title in each panel), and the blue curve is the difference between the testing tangent height minus 500 m and the testing tangent height. An error in tangent height exerts a tremendous influence on transmission. An error of 500 m in tangent height can cause a variation in transmission of 0.05 in the lower stratosphere, which can contribute to the inversion error. In the middle and upper stratosphere, the content of the atmospheric species is smaller, and the transmission is close to 1. Therefore, the transmission variation caused by a change in tangent height is small.

**Figure 8:**
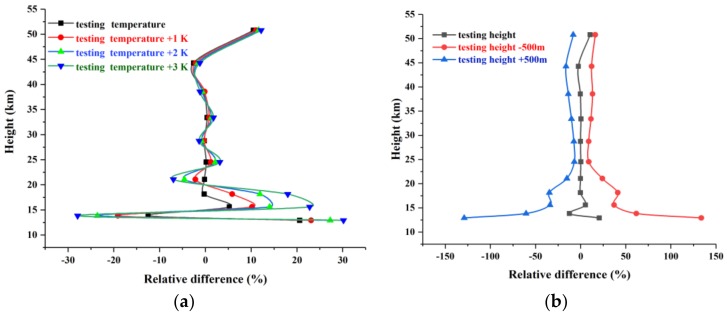
Relative differences between retrieval results and ACE-FTS NO_2_ product under different temperature errors and different tangent height errors. (**a**) Relative differences between retrieval results and ACE-FTS NO_2_ product under the conditions of exact temperature, temperature plus 1 K (red curve), temperature plus 2 K (blue curve), and temperature plus 3 K (green curve); (**b**) relative differences between retrieval results and ACE-FTS NO_2_ product under the conditions of exact tangent height, tangent height minus 500 m (red curve), and tangent height plus 500 (blue curve).

Figure 8 shows the relative differences between retrieval results and NO_2_ products of ACE-FTS under different errors in temperature and tangent heights. The errors in temperature and tangent height mainly affect the precision below the lower stratosphere (below 25 km). As can be seen from Figure 8, the impact caused by the error in tangent height (Figure 8b) is greater than the error in temperature (Figure 8a). By comparing the relative differences between relatively accurate temperature and the temperature with errors of 1 K, 2 K, and 3 K, it is found that a greater temperature leads to the greater retrieval difference, especially in the lower stratosphere. Above 25 km, the relative difference caused by the error in temperature becomes small, whereas a 1 K error in temperature causes a ~3% relative difference. An error of 500 m in tangent height can lead to ~20% retrieval error, while the retrieval relative differences are small under exact tangent heights below 25 km. However, the relative differences in the lower stratosphere (~13 km) are as great as ~140% under an error of ~500 m when the relative difference is ~10% under the relatively exact tangent height. Therefore, it is crucial to retrieve temperature profiles and correct the tangent point before the retrieval of other atmospheric species.

### 3.3. Results and Diagnostics

#### 3.3.1. Results and Diagnostics for N_2_O

**Figure 9:**
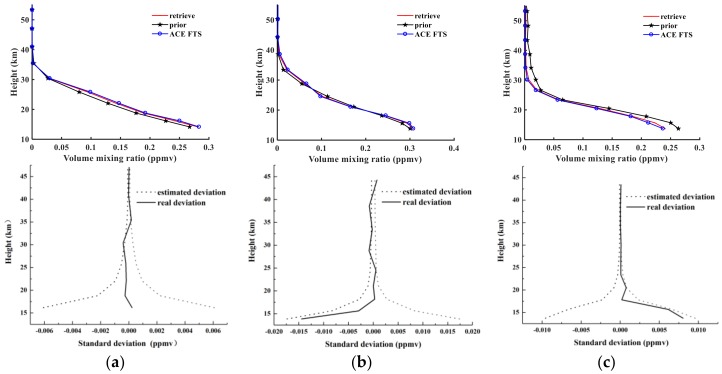
Top panels show N_2_O retrieval results for (**a**) sunrise orbit 40993; (**b**) sunrise orbit 40993; and (**c**) sunrise orbit 48359. Bottom panels show real and estimated absolute deviations for (**a**) sunrise orbit 40993; (**b**) sunrise orbit 40993; and (**c**) sunrise orbit 48359.

Figure 9 shows comparisons of three orbits of retrieval results, a priori profile, and level 2 results from ACE-FTS for N_2_O. The solid black curve is the a priori profile, the red curve shows the retrieval results of AIUS, and the blue curve represents the level 2 results for ACE-FTS. The *x*-axis is the VMR in ppmv (parts per million by volume), and the *y*-axis represents the tangent height in kilometers (km).

The top panels of Figure 9 indicate that the three orbits of N_2_O retrieval results are extremely close approximations of the ACE-FTS level 2 results and show satisfactory consistency in effective retrieval altitudes. The bottom panels of Figure 9 show a comparison between the real absolute deviations and estimated absolute deviations for sunrise orbits 40993, 43544, and 48359. Here, solid lines indicate empirical deviations that depend on the noise superimposed on the simulated measurements. At most altitudes, the deviation is less than 0.001 ppmv. The dotted curves show the estimated absolute deviations. The real absolute deviations are within the estimated deviation as evaluated using Equation (3). The displayed values are the square root of the diagonal elements of the solution covariance $S_{x}$. Furthermore, with increasing altitude, the real absolute deviations become small due to the a priori covariance matrix *S_a_*, which determines the shape of the estimated deviation. The absolute root mean standard errors (RMSEs) are 0.0056 ppmv, 0.0062 ppmv, and 0.0059 ppmv. These quantities are computed as follows:

(6)  rmsabs= [∑i=1n((xi−xacei)2)/n]12

An examination of the relative RMSE values obtained from Equation (7) is also instructive. The obtained RMSE values are 6.37%, 15.83%, and 12.59% for sunrise obits 40993, 43544, and 48359, respectively.

(7)  rmsrel= [∑i=1n(xi−xaceixacei)2/n]12

**Figure 10:**
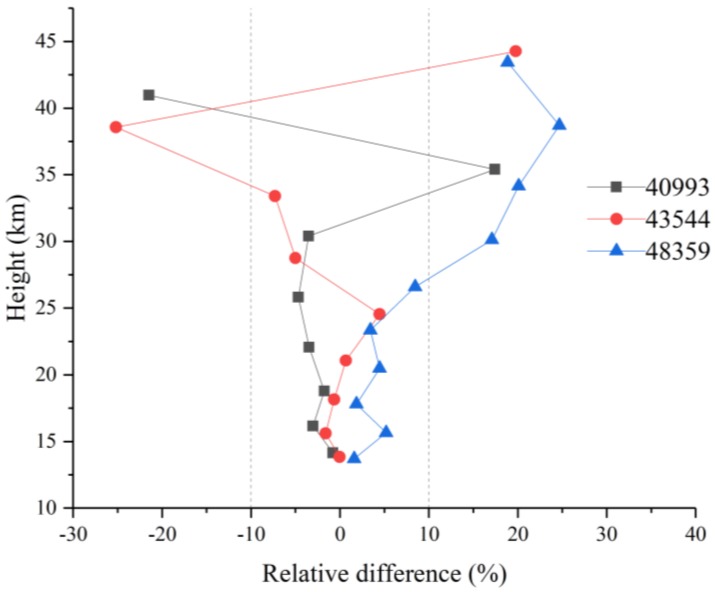
N_2_O relative differences for orbits 40993, 43544, and 48359.

In Figure 10, the relative differences between the retrieval results and the ACE-FTS level 2 results for sunrise measurements 40993, 43544, and 48359 are shown as a function of the tangent height. The solid black, red, and blue curves represent the relative differences for 40993, 43544, and 48359, respectively. The relative differences are consistent within 6% at altitudes below 25 km and between 25 and 45 km; however, the relative differences increase with altitude, with the maximum error reaching 25%. This increase occurs because of the decreasing N_2_O amount with height.

(8) $${∆}_{i}=\left(x_{i}-x_{ace_{i}}\right)/x_{ace_{i}}\cdot 100\text{\%}$$

**Figure 11:**
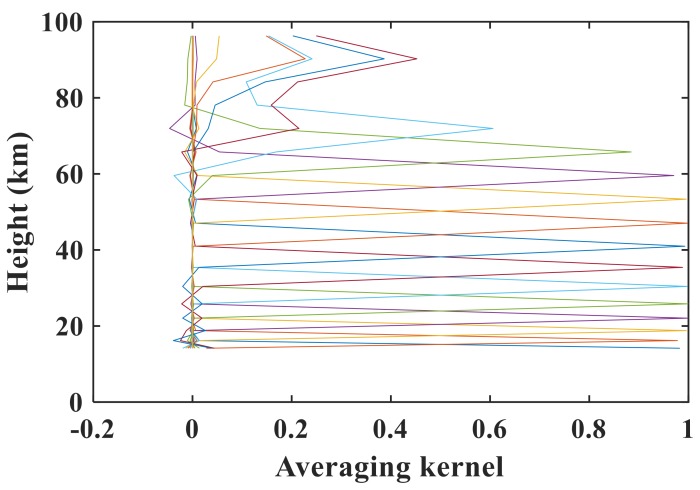
N_2_O averaging kernel for sunrise measurement 48359.

We next evaluate the influence of regularization on the retrieval results. Figure 11 shows the averaging kernel for sunrise measurement 48359 for N_2_O. Because of the similar averaging kernels for sunrise measurements 40993, 43544, and 48359, we used the averaging kernel result for sunrise measurement 48359 for each atmospheric species that we retrieved in our system as an example. The averaging kernel is approximately 0.8 at lower altitudes and exceeds 0.9 in the range of 20–50 km. The numbers of the retrieval tangent heights for the three orbits are 9, 9, and 10. According to Equation (5), the corresponding DOF values are 8.94, 8.4, and 9.37. Table 2 also lists the RMSE and DOF values for N_2_O, NO_2_, and HF for illustration. The results are close to the number of tangent heights, demonstrating that the a priori profile exerts little constraint on the retrieval results. The retrieval information originates more from the actual measurement, as can also be seen from the averaging kernel matrix. The half-width of the averaging kernel in this study is used to characterize the vertical resolution of the retrieval at the respective altitude. We find that the vertical resolution varies from 2 km at an altitude of 15 km to 5 km at an altitude of 45 km, and this result conforms to the sampling grid width.

#### 3.3.2. Results and Diagnostics for NO_2_

**Figure 12:**
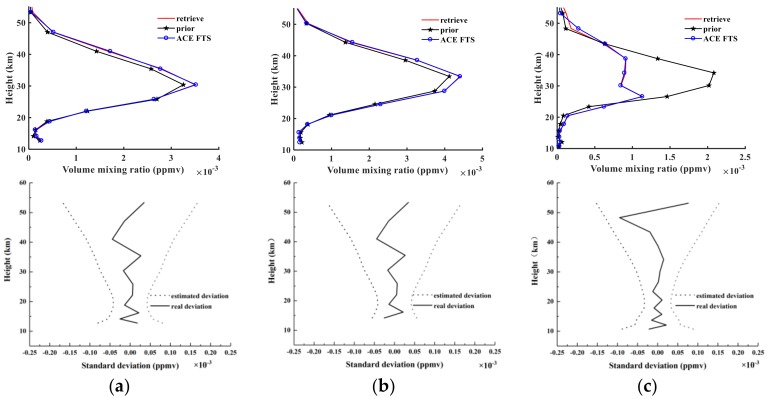
The same as Figure 9 but for NO_2_.

Figure 12 shows the retrieval results of NO_2_, the a priori profiles, and NO_2_ products from ACE-FTS level 2 for sunrise orbits 40993, 43544, and 48359. The notations used in Figure 12 are the same as in Figure 6. Figure 6a indicates that the retrieved profiles and ACE-FTS level 2 results show good agreement, corresponding to absolute RMSE values of 2.78 × 10^−5^, 2.15 × 10^−5^, and 1.32 × 10^−5^ ppmv and corresponding to relative RMSE values of 15.61%, 8.8%, and 15.87%. The atmospheric NO_2_ is distributed mainly in the stratosphere within the altitude range of 25–40 km and peaks at 30 km. The third retrieval results in Figure 12a indicate that the a priori profiles show high deviations from the ACE-FTS results, while the retrieval results fit the actual values quite well, which demonstrates that the retrieval algorithm used in the AIUS system can be quite stable. Figure 12b shows that the estimated deviations continually increase with height and that the real deviations fluctuate within the estimated deviations. The real standard deviations are less than 0.05 × 10^−3^ ppmv at almost all altitudes.

**Figure 13:**
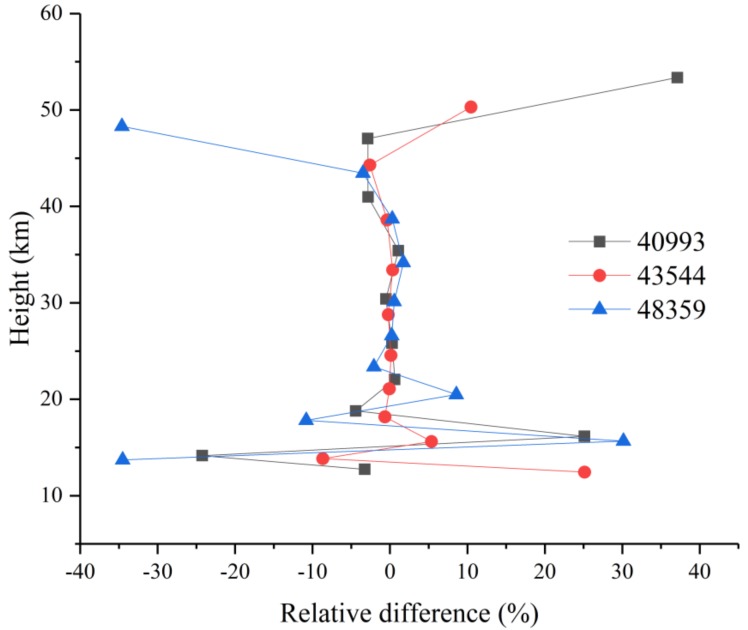
NO_2_ relative differences for orbits sr40993, sr43544, and sr48359.

Additionally, the height-correlated relative errors for NO_2_ between the retrieval results and ACE-FTS results are calculated using Equation (8) for sunrise orbits 40993, 43544, and 48359. Each line in Figure 13 has the same meaning as the corresponding line in Figure 7 but shows the data for NO_2_. In the middle and upper stratosphere, the relative differences are less than 5%. In contrast, a sudden increase in the error is observed below 20 km and above 45 km, with the maximum error reaching ±40%. The reason for this behavior is that the noise is imposed uniformly at all tangent heights and that the amount of NO_2_ in this region is smaller than the amounts present in the middle and upper stratosphere.

**Figure 14:**
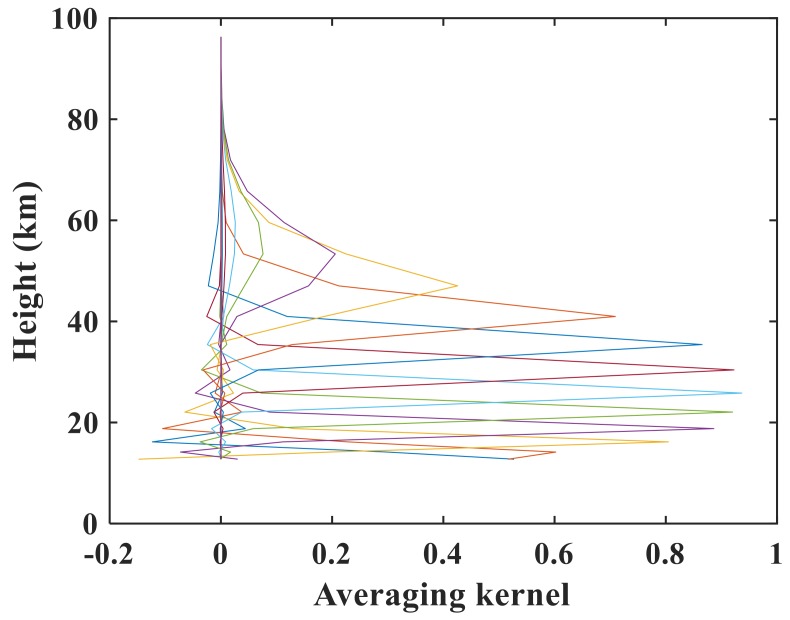
NO_2_ averaging kernel for sr48359.

The numbers of tangent heights used in NO_2_ retrieval are 10, 9, and 11 with DOFs of 6.76, 6.35, and 7.86, respectively, illustrating that NO_2_ retrieval is constrained. Figure 14 clearly shows that the averaging kernel for NO_2_ is smaller at lower altitudes and larger (0.4–06) in the range of 20–40 km (~0.9), in agreement with the distribution of the atmospheric NO_2_ content. These results imply that the retrievals at lower altitudes are constrained by the a priori profiles but that the main information in the middle and upper stratosphere comes from the measured spectra. The constraints mainly originate from lower tangent heights. The vertical resolution ranges from 2.5 km at lower altitudes to 5 km at a height of 40 km, larger than the distance between the two heights.

#### 3.3.3. Results and Diagnostics for HF

**Figure 15:**
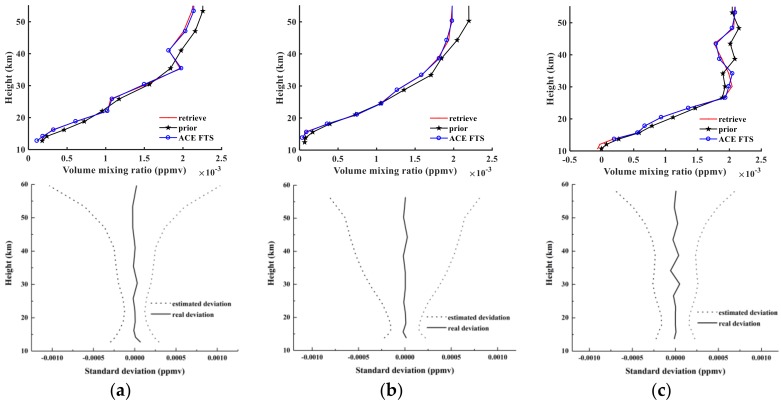
The same as Figure 9 but for HF.

Figure 15 shows the retrieval results of HF, the a priori profiles, and the HF results from ACE-FTS level 2 for sunrise orbits 40993, 43544, and 48359. The notation used in Figure 15 is the same as in Figure 9 but for HF. Relative to N_2_O and NO_2_, the retrieval results are better, and the retrieval results match the ACE-FTS results quite well because we added different noise to the measured spectra at different altitudes. The real standard deviations are also within the scope of the estimated deviation. Regardless of how the estimated deviations change, the real deviation appears to vary around a relatively small value close to 0. Using Equation (6), we obtain absolute RMSEs of 2.65 × 10^−5^, 1.44 × 10^−5^, and 2.97 × 10^−5^ ppmv, and we obtain corresponding relative RMSEs of 2.36%, 4.54%, and 2.58% according to Equation (7).

**Figure 16:**
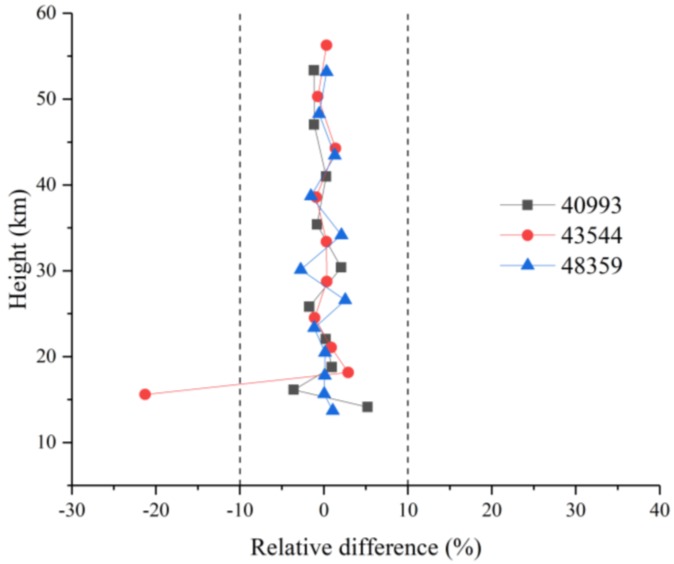
HF relative differences for sr40993, sr43544, and sr48359.

The relative differences between the HF retrieval results and ACE-FTS results are smaller (within ±5%) at all tangent heights except for one point (~−22%), as shown in Figure 16.

**Figure 17:**
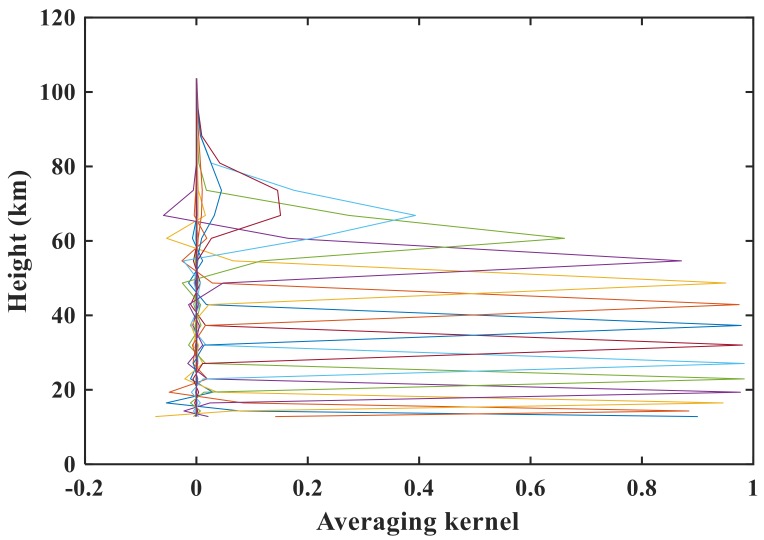
HF averaging kernel for sr48359.

Figure 17 indicates that the maximum averaging kernel is as great as 0.95 and that the minimum also exceeds 0.9 for 15 km and 55 km, which is in accordance with the results for the number of DOFs. The numbers of the three retrieval tangent heights are 8, 9, and 10 with DOFs of 7.7, 8.11, and 9.29, respectively, as calculated by Equation (5). Similar to N_2_O, the retrieval results are influenced by the observed spectra rather than by the a priori spectra, as shown by the averaging kernel. The vertical resolution at lower altitudes can reach 1.5 km and decreases with increasing altitudes. The resolution calculated from the averaging kernel is identical to the spacing between two contiguous tangent heights, being even finer than the grid width used in the AIUS system.

## 4. Statistical Analysis

**Figure 18:**
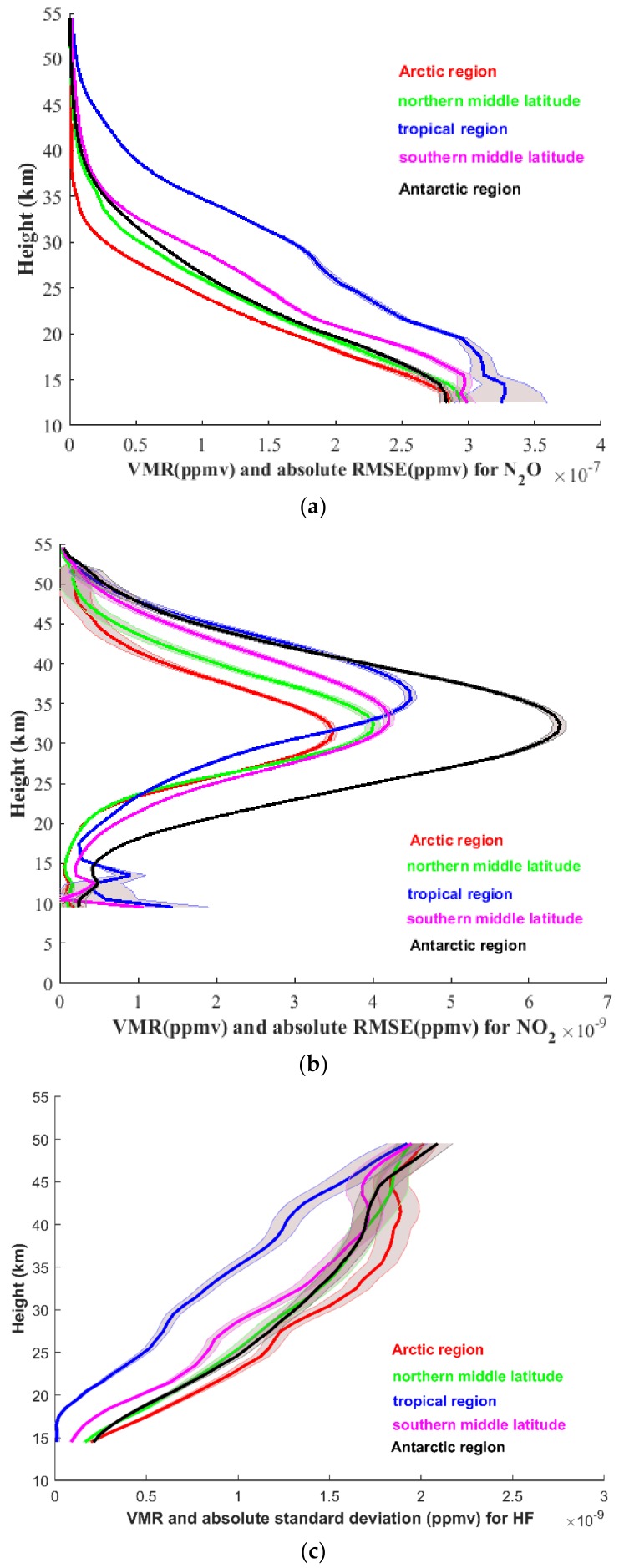
Mean volume mixing ratio (VMR) profiles and absolute standard deviations in five regions. (**a**) For N_2_O; (**b**) for NO_2_; (**c**) for HF.

To further assess the simulation retrieval algorithm, a large number of retrieval tests were performed for each component in May 2018. To verify the generalizability of the inversion algorithm in the global region, the selected experimental areas cover the Arctic region (~75° N), northern middle latitude (~45° N), tropics (~5° N), southern middle latitude (~45° S), and Antarctic region (~75° S). Hundreds of retrievals were conducted separately in each region related to N_2_O, NO_2_, and HF species, and both the VMR profiles and the absolute standard deviations in each region were all averaged. Figure 18 lists the mean VMR profiles of the retrieval results and the averaged absolute standard deviations. Each curve of a specific color represents a different region (the red curve represents the Arctic region, the green represents the northern middle latitude, the blue is the tropical region, the magenta is the southern middle latitude, and the black is the Antarctic region). Overall, the trends of the mean VMR profiles and the averaged absolute standard deviations of the three species (N_2_O, NO_2_, and HF) are consistent with the results in Section 3. However, great variation in mean VMR profiles occurs in each region.

The VMR of N_2_O generally increases with increasing altitude, as shown in Figure 18a. The averaged absolute standard deviations are 0.1 × 10^−7^ ppmv, except in the lower altitude (below 20 km, with a maximum value of ~0.5 × 10^−7^ ppmv) of the tropical region. The VMR of N_2_O is larger in the tropics than in the other four regions, and the minimum VMR of N_2_O is in the Antarctic region.

Figure 18b shows the results for NO_2_. The averaged absolute standard deviations of NO_2_ show a low standard deviation in the stratosphere and relatively higher standard deviations below 15 km and above 45 km. The maximum VMR of NO_2_ exists in the Antarctic region, and the minimum occurs in the Arctic region. This characteristic differs from the VMR profiles of N_2_O.

The VMR of HF in the bottom panel of Figure 18 increases with altitude, and the value is larger in the Arctic region and the Antarctic region than in the other regions, whereas the tropical region has the minimum VMR of HF. Compared with N_2_O and NO_2_, the absolute standard deviation of HF is relatively larger in the altitude range of 25–50 km, with a maximum absolute standard deviation of 10^−7^ ppmv at the altitude of 40 km in the Arctic region. According to the results of retrieving quantities of profiles and computing the absolute standard deviation, the AIUS system can generally maintain stability and ensure precision.

## 5. Conclusions

This study employs the OEM and RFM to retrieve three orbits of N_2_O, NO_2_, and HF profiles. The overall absolute and relative RMSE, the relative errors at different tangent heights between the retrieval results, and the ACE-FTS products are calculated to analyze the differences. To accurately characterize the retrieval algorithm, we also calculate the estimated deviation and real deviation. Finally, several diagnostic tools, such as the vertical resolution, averaging kernel, and number of DOFs, are used to assess the inversion model of AIUS. Before the analysis of retrieval results, the impacts on the retrieval results caused by errors in temperature and tangent height are discussed. The errors in temperature and tangent height mostly affect the precision below the lower stratosphere. The analysis results are as follows.

For the N_2_O retrieval results, the absolute RMSE values of the three orbits are 0.0056, 0.0062, and 0.0059 ppmv, and the corresponding relative RMSE values are 6.37%, 25.83%, and 12.59%. The relative errors are smaller than 6% at altitudes below 25 km and are larger in the 25–45 km range, with a maximum value of 25%. At most altitudes, the absolute real deviation is less than 0.001 ppmv, which is within the estimated deviation. The N_2_O retrieval information is mainly derived from the observed spectra, not from the a priori profiles.

For NO_2_, when the a priori profiles show large deviations from the ACE-FTS results and the shape is different, the retrieval results can also fit the actual values quite well. The absolute RMSE values are 2.78 × 10^−5^, 2.15 × 10^−5^, and 1.32 × 10^−5^ ppmv, and the relative RMSE values do not exceed 5% in the middle and upper stratospheres. Even though the estimated deviations increase continuously with height, the real deviations fluctuate within the estimated deviations. The inversion for NO_2_ is constrained by the a priori profile below 20 km and above 45 km.

We obtain better HF retrieval profiles with absolute RMSE values of 2.65 × 10^−5^, 4.54 × 10^−5^, and 2.97 × 10^−5^ ppmv and corresponding relative RMSE values of 2.36%, 14.54%, and 2.58%, except for one tangent point of sunrise orbit 43544. Almost all the relative errors are controlled within 5%, and the real absolute deviations vary around a relatively small value. Similar to N_2_O, the retrieval results are influenced by the observed spectra.

Finally, the retrieval method is applied in five regions in the global region. For each species, one hundred retrieval tests are performed, and a preliminary analysis of the VMR profiles and the standard deviation is performed. The trends of the mean VMR profiles and the averaged absolute standard deviations of the N_2_O, NO_2_, and HF are consistent with the results in Section 3. However, great variation in mean VMR profiles occurs in each region. The VMR of N_2_O is larger in the tropics than in the other four regions, and the minimum VMR of N_2_O is in the Antarctic region. With a different characteristic from the VMR profiles of N_2_O, the maximum VMR of NO_2_ exists in the Antarctic region, and the minimum occurs in the Arctic region. The VMR of HF is larger in the Arctic region and the Antarctic region than in the other regions, whereas the tropical region has the minimum VMR of HF. Generally, the AIUS system can maintain stability, and precision can be ensured.

In our AIUS retrieval system, we use reliable a priori knowledge for the initial estimations in the inversion model and integrate atmospheric profiles for the forward model, and the obtained results meet our expectations. However, the retrieval process is based entirely on the ACE-FTS data rather than on the AIUS real measurement data. Currently, AIUS is in an in-orbit test period; therefore, all of these results will be recalculated using the real measurement data at a later date.

## Figures and Tables

**Table 1:** Comparisons of number of channels and information content between the Atmospheric Chemistry Experiment–Fourier Transform Spectrometer (ACE-FTS) and simulating the Atmospheric Infrared Ultraspectral Sounder (AIUS).

		ACE-FTS	Simulating AIUS
N_2_O	NC	2040	184
	IC	6.92	6.10
NO_2_	NC	1384	126
	IC	7.38	6.69
HF	NC	485	283
	IC	6.84	6.43

**Table 2:** RMSE and DOFs for N_2_O, NO_2_, and HF retrieval results.

	Orbits	sr40993	sr43544	sr48359
RMSE and DOF				
N_2_O	A-RMSE (ppmv)	0.0056	0.0062	0.0059
	R-RMSE (%)	6.37%	15.83%	12.59%
	NTH	9	9	10
	DOF	8.94	8.4	9.37
NO_2_	A-RMSE (ppmv)	2.78 × 10^−5^	2.15 × 10^−5^	1.32 × 10^−5^
	R-RMSE (%)	15.61%	8.8%	15.87%
	NTH	10	9	11
	DOF	6.76	6.35	7.86
HF	A-RMSE (ppmv)	2.65 × 10^−5^	1.44 × 10^−5^	2.97 × 10^−5^
	R-RMSE (%)	2.36%	4.54%	2.58%
	NTH	8	9	10
	DOF	7.7	8.11	9.29

Note: A-RMSE is the absolute root mean standard error, R-RMSE is the relative root mean standard error, NTH is the number of tangent heights used in the retrieval process, and DOFs is the number of degrees of freedom.

## References

[1] Li X., Cheng T., Xu J., Shi H., Zhang X., Ge S., Zou M., Wang H., Wang Y., Zhu S. (2018). Trace Gas Retrieval from AIUS: Algorithm Description and O_3_ Retrieval Assessment. Preprints.

[2] Bernath P. (2006). Atmospheric Chemistry Experiment (ACE): Analytical Chemistry from Orbit. Trends Anal. Chem..

[3] Kerzenmacher T., Wolff M.A., Strong K., Dupuy E. (2008). Validation of NO_2_ and NO from the Atmospheric Chemistry Experiment (ACE). Atmos. Chem. Phys. Discuss..

[4] Holland E.A., Braswell B.H., Sulzman J., Lamarque J.F. (2005). Nitrogen deposition onto the United States and Western Europe: Synthesis of observations and models. Ecol. Appl..

[5] Strong K., Wolff M.A., Kerzenmacher T.E., Walker K.A., Bernath P.F., Blumenstock T., Boone C., Catoire V., Coffey M., de Mazière M. (2008). Validation of ACE-FTS N_2_O measurements. Atmos. Chem. Phys..

[6] Chipperfield M.P., Burton M., Bell W., Walsh C.P., Blumenstock T., Coffey M.T., Hannigan J.W., Mankin W.G., Galle B., Mellqvist J. (1997). On the use of HF as a reference for the comparison of stratospheric observations and models. J. Geophys. Res. Atmos..

[7] Gunson M.R., Abbas M.M., Abrams M.C., Allen M., Brown L.R., Brown T.L., Chang A.Y., Goldman A., Irion F.W., Lowes L.L. (1996). The Atmospheric Trace Molecule Spectroscopy (ATMOS) experiment: Deployment on the ATLAS Space Shuttle missions. Geophys. Res. Lett..

[8] Gunson M.R., Farmer C.B., Norton R.H., Zander R., Rinsland C.P. (1990). Measurements of CH_4_, N_2_O, CO, H_2_O, and O_3_ in the middle atmosphere by the Atmospheric Trace Molecule Spectroscopy Experiment on Spacelab 3. J. Geophys. Res..

[9] Russell J.M., Gordley L.L., Park J.H., Drayson S.R., Hesketh W.D., Cicerone R.J., Tuck A.F., Frederick J.E., Harries J.E., Crutzen P.J. (1993). The Halogen Occultation Experiment. J. Geophys. Res.-Atmos..

[10] Gordley L.L., Iii J.M.R., Mickley L.J., Frederick J.E., Park J.H., Stone K.A., Beaver G.M., McInerney J.M., Deaver L.E., Toon G.C. (1996). Validation of nitric oxide and nitrogen dioxide measurements made by the Halogen Occultation Experiment for UARS platform. J. Geophys. Res. Atmos..

[11] Iii J.M.R., Deaver L.E., Luo M., Cicerone R.J., Park J.H., Gordley L.L., Toon G.C., Gunson M.R., Traub W.A., Johnson D.G. (1996). Validation of hydrogen fluoride measurements made by the Halogen Occultation Experiment from the UARS platform. J. Geophys. Res..

[12] Mahieu E., Duchatelet P., Demoulin P., Walker K.A., Dupuy E., Froidevaux L., Randall C., Catoire V., Strong K., Boone C.D. (2008). Validation of ACE-FTS v2.2 measurements of HCl, HF, CCl_3_F and CCl_2_F_2_ using space-, balloon- and ground-based instrument observations. Atmos. Chem. Phys. Discuss..

[13] Boone C.D., Nassar R., Walker K.A., Rochon Y., McLeod S.D., Rinsland C.P., Bernath P.F. (2005). Retrievals for the atmospheric chemistry experiment Fourier-transform spectrometer. Appl. Opt..

[14] Fischer H., Birk M., Blom C., Carli B., Carlotti M., von Clarmann T., Delbouille L., Dudhia A., Ehhalt D., Endemann M. (2008). MIPAS: An instrument for atmospheric and climate research. Atmos. Chem. Phys..

[15] Beer R., Glavich T.A., Rider D.M. (2001). Tropospheric emission spectrometer for the Earth Observing System’s Aura Satellite. Appl. Opt..

[16] Beer R. (2006). TES on the Aura mission: Scientific objectives, measurements, and analysis overview. IEEE Trans. Geosci. Remote Sens..

[17] Clarmann T.V., Höpfner M., Kellmann S., Linden A., Chauhan S., Funke B., Grabowski U., Glatthor N., Kiefer M., Schieferdecker T. (2009). Retrieval of temperature, H_2_O, O_3_, HNO_3_, CH_4_, N_2_O, ClONO_2_ and ClO from MIPAS reduced resolution nominal mode limb emission measurements. Atmos. Meas. Tech. Discuss..

[18] Rodgers C.D. (1976). Retrieval of atmospheric temperature and composition from remote measurements of thermal radiation. Rev. Geophys. Space Phys..

[19] Rodgers C.D. (2000). Inverse Methods for Atmospheric Sounding: Theory and Practice.

[20] Eriksson P. (2000). Analysis and comparison of two linear regularization methods for passive atmospheric observations. J. Geophys. Res..

[21] Eriksson P., Jime′neza C., Buehler S.A. (2005). Qpack, a general tool for instrument simulation and retrieval work. J. Quant. Spectrosc. Radiat. Transf..

